# BQ123 Stimulates Skeletal Muscle Antioxidant Defense via Nrf2 Activation in LPS-Treated Rats

**DOI:** 10.1155/2016/2356853

**Published:** 2015-12-28

**Authors:** Agata Kowalczyk, Agnieszka Jeleń, Marta Żebrowska, Ewa Balcerczak, Anna Gorąca

**Affiliations:** ^1^Department of Cardiovascular Physiology, Chair of Experimental and Clinical Physiology, Medical University of Lodz, 6/8 Mazowiecka Street, 92-215 Lodz, Poland; ^2^Laboratory of Molecular Diagnostic and Pharmacogenomics, Department of Pharmaceutical Biochemistry and Molecular Diagnostic, Medical University of Lodz, 1 Muszynskiego Street, 90-151 Lodz, Poland

## Abstract

Little is understood of skeletal muscle tissue in terms of oxidative stress and inflammation. Endothelin-1 is an endogenous, vasoconstrictive peptide which can induce overproduction of reactive oxygen species and proinflammatory cytokines. The aim of this study was to evaluate whether BQ123, an endothelin-A receptor antagonist, influences the level of TNF-*α*, IL-6, SOD-1, HO-1, Nrf2 mRNA, and NF-*κ*B subunit RelA/p65 mRNA in the femoral muscle obtained from endotoxemic rats. Male Wistar rats were divided into 4 groups (*n* = 6) and received *iv* (1) saline (control), (2) LPS (15 mg/kg), (3) BQ123 (1 mg/kg), (4) BQ123 (1 mg/kg), and LPS (15 mg/kg, resp.) 30 min later. Injection of LPS led to significant increase in levels of RelA/p65 mRNA, TNF-*α*, and IL-6, while content of SOD-1, HO-1, and Nrf2 mRNA was unchanged. Administration of BQ123 prior to LPS challenge resulted in a significant reduction in RelA/p65 mRNA, TNF-*α*, and IL-6 levels, as well as markedly elevated concentrations of SOD-1, HO-1, and Nrf2 mRNA. BQ123 appears to enhance antioxidant defense and prevent production of TNF-*α* and IL-6 in skeletal muscle of LPS-treated rat. In conclusion, endothelin-A receptor antagonism exerts significant impact on the skeletal muscle favouring anti-inflammatory effects and protection against oxidative stress.

## 1. Introduction

Sepsis is a severe systemic inflammation contributing to excessive generation of reactive oxygen species (ROS), overproduction of numerous inflammatory cytokines, and multiple organ failure, which often results in death [1]. This critical condition is a frequent cause of such neuromuscular disorders as critical illness myopathy (CIM), which may lead to rhabdomyolysis and muscle atrophy [2]. Lipopolysaccharide (LPS), the main causative agent inducing sepsis, stimulates macrophages to excrete large amounts of inflammatory biomarkers, for example, tumour necrosis factor-*α* (TNF-*α*), interleukin-1 (IL-1), IL-6, and IL-8. [1, 3]. High serum levels of TNF-*α* and IL-6 accompanying endotoxemia are believed to induce protein degradation in skeletal muscle contributing to muscular atrophy [4]. The appearance of these mediators in blood is in mostly the effect of activation of the nuclear factor-*κ*B (NF-*κ*B) pathway, a key regulator of immune system response [1, 3]. NF-*κ*B is present in almost every mammalian cell, located as a heterodimer consisting of two subunits, p50 and RelA/p65. Under the influence of such factors as LPS and TNF-*α*, NF-*κ*B translocates to the nucleus, where it initiates expression of inflammatory cytokines and the adhesion molecules involved in proliferation, apoptosis, and oxidative stress response [5].

The deleterious participation of ROS in myopathy has been studied by many authors [6, 7]. ROS accumulation, especially mitochondrial ROS, has been shown to play a significant role in muscle atrophy [7]. ROS can cause DNA damage, lipid peroxidation, and protein modification and may activate certain nuclear transcription factors such as NF-*κ*B [8] and nuclear factor (erythroid-derived-2)-like 2 (Nrf2) [9]. Linke et al. indicated in skeletal muscle reduced activity of major antioxidant enzymes, that is, superoxide dismutase (SOD), catalases (CAT), and glutathione peroxidase (GPX) during oxidative stress [10]. Treatment with SOD and CAT or supplementation with antioxidant vitamin attenuated oxidative stress and skeletal muscle atrophy [11, 12].

Many authors demonstrated that endothelin-1 (ET-1), a vasoconstrictive, endothelial peptide, accelerates ROS formation in vascular smooth muscle cells (VSMC), endothelial cells, and other tissues [13–15]. Skeletal muscle is one of the most vascularized tissues [16], but little is known about ET-1 participation in the development of oxidative stress in this tissue. What is more, LPS is known to increase endothelial permeability and intensify production of ET-1 in various tissues [17, 18]. Piechota et al. indicate that ET-1 levels are correlated with other parameters of sepsis such as C-reactive protein, procalcitonin, or natriuretic propeptide [19], implying that ET-1 is involved in pathogenesis of sepsis and blood levels of ET-1 may serve as a biomarker of severity of sepsis [20, 21]. Endothelin-1 acts through two types of G protein-coupled receptors, endothelin receptor A (ETA) and endothelin receptor B (ETB), both of which are present in multiple various cells and tissues. Under physiological conditions, ET-1 binding to the ETA receptors on VSMC triggers a potent vascular smooth muscle contraction [22], while a high level of ET-1 additionally results in intensified synthesis of ROS, mainly superoxide anion (O_2_^∙−^) [23]. Blockage of the ETA receptor with BQ123, a receptor antagonist, decreases the content of lipid peroxidation products [24, 25], alleviates LPS-induced oxidative stress [15, 26, 27], increases reduced glutathione (GSH) level, and enhances SOD activity [25, 28]. Virtually, no reports describe the influence of BQ123 on the Nrf2/heme oxygenase-1 (HO-1) signaling pathway.

Nrf2 is a crucial agent regulating the expression of antioxidant/detoxification genes encoding many cytoprotective proteins that act in synergy to remove ROS [29]. Under normal conditions, Nrf2 is found in the cytoplasm, coupled with the regulatory protein Keap1 [30]. In response to oxidative stress, Nrf2 dissociates from this complex and transfers to the nucleus, where it binds to the specific ARE sequence and upregulates the expression of antioxidant genes such as HO-1, which plays an important role protecting against oxidative stress and inflammatory processes [31]. Some authors suggest that potential cross talk may exist between Nrf2 and NF-*κ*B pathways [30].

The present study investigates the influence of BQ123 on inflammatory process (RelA/p65 mRNA, TNF-*α*, and IL-6 levels) and antioxidant response (Nrf2 mRNA, HO-1, and SOD-1 levels) in the skeletal muscle of endotoxemic rats.

## 2. Materials and Methods

### 2.1. Animals

All experiments were carried out on male Wistar rats aged 3-4 months, weighing 270–330 g: the rats were weighed directly before the experiment. The animals were kept under standard laboratory temperature (20 ± 2°C) and lighting (light from 6:00 a.m. to 6:00 p.m.), with free access to lab chow and tap water, until being used in the experiments. All animals were maintained for 1 week in the laboratory for adaptation. The experimental procedures followed the guidelines for the care and use of laboratory animals and were approved by the Medical University of Lodz Ethics Committee number 7/ŁB699/2014.

### 2.2. Experimental Protocol

Animals were randomly divided into four groups (*n* = 6 per group). In group 1 (control), rats received* iv *0.2 mL of 0.9% NaCl and 30 min later again 0.2 mL of 0.9% NaCl. In group 2 (LPS), rats received* iv *0.2 mL of saline and 30 min later 0.2 mL of LPS (15 mg/kg). In group 3 (BQ123), rats received* iv *0.2 mL of saline and 30 min later 0.2 mL of BQ123 (1 mg/kg). In group 4 (BQ123 + LPS), rats received* iv* a single dose of BQ123 (1 mg/kg) and a single dose of LPS (0.2 mL, 15 mg/kg) after 30 min. The animals were anesthetized by an intraperitoneal injection of urethane solution (1.5 g/kg of b.w.). When a sufficient level of anesthesia was achieved, the skin and subcutaneous tissues on the neck were infiltrated with 2% lidocaine hydrochloride solution (Polfa, Poland) and cut and a 2 cm-long polyethylene tube (2.00 mm O.D.) was inserted into the trachea. The right femoral vein was catheterized and a polyurethane cannula was inserted (0.41 mm O.D., 0.23 mm I.D.). All drugs were administrated directly into the femoral vein.

### 2.3. Tissue Preparation and Sample Collection

Five hours after the last injection, the rats were sacrificed. The femoral muscle was cut off at the right thigh and rinsed with ice-cold saline, dried by blotting between two pieces of filter paper, weighed, and frozen in −75°C until being used for measurements.

### 2.4. Determination of TNF-*α*, IL-6, and SOD-1 Levels

TNF-*α*, IL-6, and SOD-1 concentrations in the skeletal muscle were assayed by specific enzyme linked immunosorbent assay using a commercially available ELISA test kit containing a monoclonal antibody specific for rat TNF-*α*, IL-6, and SOD-1 (Cloud-Clone Corp., USA). Firstly, 50 mg portions of skeletal muscle were cut into small pieces and homogenized in 2 mL of ice-cold PBS with a glass homogenizer on ice. The resulting suspension was subjected to two freeze-thaw cycles to further break the cell membranes. The homogenates were centrifuged for 5 min at 5000 ×g in 4°C, and the supernatants were collected and assayed immediately according to the manufacturer's instructions. Optical density at 450 nm was read using Victor x3 microplate reader (Perkin Elmer, USA). All tests were performed in duplicate. Protein concentration of the samples was determined using the Bio-Rad Protein Assay (Bio-Rad Laboratories, USA), according to the manufacturer's instructions. The TNF-*α* and IL-6 concentrations were expressed as pg/mg protein. The concentration of SOD-1 was expressed as ng/mg protein.

### 2.5. Determination of HO-1 Level

An ELISA kit (Enzo Life Sciences, Cat. number ADI-EKS-810A) was used to evaluate the concentration of HO-1. Firstly, 50 mg portions of skeletal muscle were cut into small pieces and homogenized in 1 mL of extraction reagent with the addition of protease inhibitors. Tissues were homogenized in glass homogenizer on ice. The homogenates were centrifuged at 21,000 ×g in 4°C for 10 min. Supernatants were removed and assayed immediately according to the manufacturer's instructions. Optical density was read at 450 nm using a Victor x3 microplate reader (Perkin Elmer, USA). All tests were performed in duplicate. Protein concentration of the samples was determined using the Bio-Rad Protein Assay (Bio-Rad Laboratories, USA), according to the manufacturer's instructions. The HO-1 concentration was expressed as ng/mg protein.

### 2.6. RNA Isolation

Total RNA was extracted from samples using RNeasy mini kits (Qiagen). Briefly, frozen samples of rat femoral muscle were homogenized in 300 *μ*L of RLT Buffer by Tissue Ruptor homogenizer (Qiagen). Then, 590 *μ*L of Nuclease-Free Water (Ambion) and 10 *μ*L of Qiagen Proteinase K solution were added. Homogenates were incubated at 55°C for 10 min and centrifuged for 3 min at 14000 rpm. The following part of protocol was performed as described by the manufacturer. RNA was quantified using a Pico Drop spectrophotometer (Picodrop Limited, UK). The quality of RNA samples was analyzed by measuring the ratio of absorptions at 260/280 nm. The purified total RNA was immediately used for cDNA synthesis or stored at −80°C. Generation of cDNA was performed with QuantiTect Reverse Transcription Kit (Qiagen) according to the protocol of the manufacturer, with 1 *μ*g of total RNA used as starting material. Reverse transcription was performed in conditions optimized for use with this kit (25°C for 10 min, 37°C for 120 min, and 85°C for 5 min). The cDNA samples were kept frozen at −20°C.

### 2.7. Determination of Nrf2 and p65 mRNA Expressions: Real Time PCR Analysis

The mRNA quantification was done using standard TaqMan Gene Expression Assays (Applied Biosystems), Nfe2l2 (Assay ID: Rn00477784_m1), Rela (Assay ID: Rn01502266_m1), and Actb (Rn00667869_m1), as a control. The 20 *μ*L qPCR included 50 ng cDNA, 10 *μ*L TaqMan Universal PCR Master Mix, and 1 *μ*L TaqMan Gene Expression Assay (20x). The reactions were incubated in a 96-well plate at 95°C for 10 min, followed by 40 cycles of 95°C for 15 s and 60°C for 1 min. All reactions were run in triplicate. TaqMan PCR assays were performed on a 7900HT Fast Real-Time PCR System (Applied Biosystems) and analyzed using Sequence Detection System 2.3 Software. Fold induction values (RQ) were calculated according to the equation 2^−ΔΔCt^, where ΔCt represents the differences in cycle threshold numbers between the target gene (Nrf2, RelA) and endogenous control (*β*-actin) and ΔΔCt represents the relative change in these differences between examined and control groups.

### 2.8. Statistical Analysis

STATISTICA 12 (StatSoft) program was used to perform statistical calculations. The results were presented as means ± SEM. Statistical analyses of the difference between two groups were performed using independent Student's* t*-test. Values of *p* < 0.05 were accepted as statistically significant.

## 3. Results

### 3.1. BQ123 Pretreatment Lessens LPS-Induced Production of Inflammatory Biomarkers

**Figure 1:**
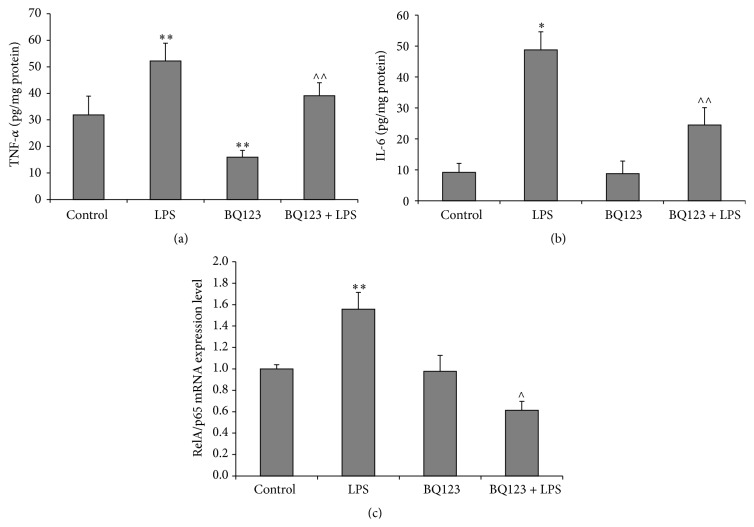
The effect of LPS (15 mg/kg), BQ123 (1 mg/kg), and BQ123 + LPS (1 mg/kg and 15 mg/kg, resp.) on TNF-*α* (a), IL-6 (b), and RelA/p65 mRNA (c) levels in the rat skeletal muscle. Results are expressed as mean ± SEM. *n* = 6 per group. ^*∗*^ *p* < 0.001 versus control; ^*∗∗*^ *p* < 0.01 versus control; ^∧^ *p* < 0.001 versus LPS; ^∧∧^ *p* < 0.01 versus LPS.

Skeletal muscle levels of TNF-*α* and IL-6 are illustrated in Figures 1(a) and 1(b). LPS treatment led to increased tissue levels of TNF-*α* and IL-6 when compared to the control group (*p* < 0.01 and *p* < 0.001, resp.). Concomitant treatment with BQ123 significantly decreased the LPS-induced production of these cytokines as compared to the LPS group (*p* < 0.01). Moreover, BQ123 applied alone resulted in lowered level of TNF-*α* as compared to the control (*p* < 0.01).

### 3.2. BQ123 Administration Alters the Expression of Nrf2 and RelA/p65 mRNA during Endotoxemia

**Figure 2:**
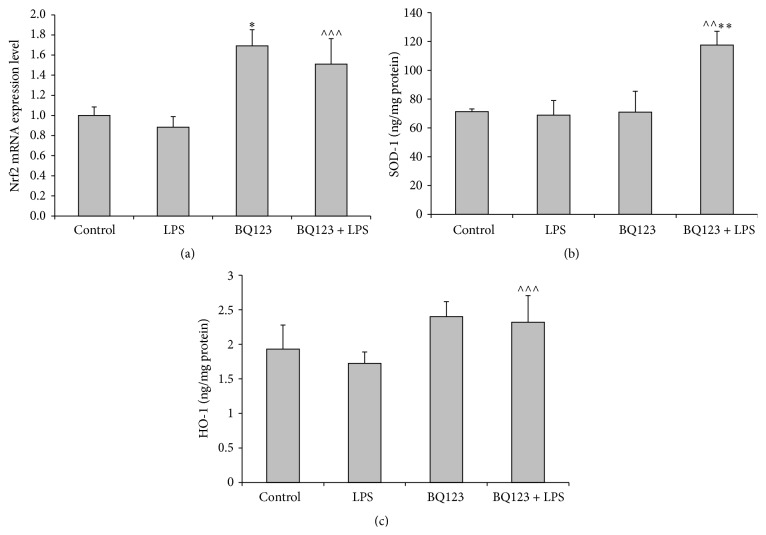
The effect of LPS (15 mg/kg), BQ123 (1 mg/kg), and BQ123 + LPS (1 mg/kg and 15 mg/kg, resp.) on Nrf2 mRNA (a), HO-1 (b), and SOD-1 (c) levels in the rat skeletal muscle. Results are expressed as mean ± SEM. *n* = 6 per group. ^*∗*^ *p* < 0.001 versus control; ^*∗∗*^ *p* < 0.01 versus control; ^∧∧^ *p* < 0.01 versus LPS; ^∧∧∧^ *p* < 0.05 versus LPS.

Figures 1(c) and 2(a) present RelA/p65 and Nrf2 mRNA expression levels in the rat skeletal muscle. RelA/p65 mRNA expression is significantly increased in LPS group when compared to the control (*p* < 0.01), while Nrf2 mRNA level is slightly and insignificantly decreased. However, administration of BQ123 alone successfully activated the expression of Nrf2 (*p* < 0.001) compared to the control but did not affect RelA/p65 mRNA level. Otherwise, injection of BQ123 followed by LPS significantly elevated expression of Nrf2 (*p* < 0.05) as compared to the LPS group, whereas RelA/p65 expression in the same group was substantially declined (*p* < 0.001).

### 3.3. BQ123 Pretreatment Enhanced Antioxidant Defense during Endotoxemia

ELISA results showed that concentration of SOD-1 was unchanged in the LPS group when compared to the control (Figure 2(b)). However, pretreatment with BQ123 resulted in increased SOD-1 levels compared with the LPS group (*p* < 0.01) and the control (*p* < 0.01). Concentration of HO-1 in LPS and BQ123 groups was slightly different from the control group and showed no statistical significance (Figure 2(c)). However, the HO-1 level in BQ123 + LPS group turned out to be increased (*p* < 0.05).

## 4. Discussion

Our present findings are the first to demonstrate that BQ123, an ETA receptor blocker, increases expression of Nrf2 in femoral muscle of LPS-treated rats. The increased expression of Nrf2 was associated with enhanced levels of SOD-1 and HO-1 and decreased production of TNF-*α* and IL-6 in skeletal muscle.

It is well documented that LPS leads to the development of inflammation associated with oxidative stress that can cause tissue damage, including skeletal muscle damage [32–34]. The markedly elevated concentrations of TNF-*α* and IL-6 in muscle tissue found in the present study after LPS challenge confirm the results of other authors, who demonstrated the same effect of LPS in rat skeletal muscle [35] and in L6 skeletal muscle cells [36]. Moreover, Olesen et al. observe that LPS injection (0.3 ng/kg b.w.) increased TNF-*α* and IL-6 mRNA content in the skeletal muscle of young, male volunteers [37]. LPS and ROS are well known to induce migration of NF-*κ*B to the nucleus, where NF-*κ*B stimulates the expression of proinflammatory genes. NF-*κ*B activates TNF-*α* and IL-6 production, which in turn stimulates nuclear translocation of NF-*κ*B, forming a loop feedback mechanism [38]. This was demonstrated by the substantially higher level of RelA/p65 mRNA observed in the present study in rats receiving LPS alone.

It was reported that the ETA receptor blockade reduces levels of oxidative stress parameters [39–41]. Several studies have shown that BQ123 has a beneficial influence on the TNF-*α* level in the lung tissue of rats treated with cigarette smoke extract [42], in rat hearts with ischemia-reperfusion injury [28], and in patients after bypass grafting [43]. This ETA receptor antagonist also alleviates IL-6 production in human vascular smooth muscle cells [44]. The beneficial effects of BQ123 are probably associated with the inhibition of NF-*κ*B expression observed in our present study through significant reduction of RelA/p65 mRNA, the NF-*κ*B subunit, in LPS-treated rats. Pretreatment with BQ123 was also found to decrease LPS-elicited augmentation of TNF-*α* and IL-6, confirming that endothelin-1 mediates the activation of the NF-*κ*B pathway and blocking ETA receptor exhibits an anti-inflammatory effect.

To avoid harmful effects triggered by ROS and inflammatory cytokines, the skeletal muscles control the antioxidant defense system, which includes the enzymatic antioxidants such as HO-1, SOD, CAT, and GPX and the nonenzymatic free radical scavengers, that is, glutathione, thioredoxin [45, 46]. The main activity of HO-1 is to metabolize heme to iron, carbon monoxide (CO), and biliverdin, which is immediately converted into bilirubin. It is an antioxidative phase II enzyme since the products of heme degradation have antiradical, anti-inflammatory, and antiapoptotic properties [47]. In addition, overexpression of HO-1 can negatively regulate inflammatory mediators, including TNF-*α* and IL-6 [48, 49]. Our findings indicate that LPS administration resulted in slightly lower level of HO-1 than control values. Likewise, Tran et al. report a nonsignificant decrease in HO-1 after LPS stimulation in murine BV2 microglia cell line [50]. On the other hand, Wang et al. indicate a decrease in HO-1 protein expression in the aorta of rats treated* iv* with 10 mg/kg LPS [51]. Similar results were presented by Seo et al. following 6 hours of LPS exposure in the RAW 264.7 macrophage cell line [52]. However, other authors demonstrated increase in HO-1 level in the diaphragmatic muscle after LPS stimulation. Barreiro et al. reported enhanced HO-1 protein expression within 6–24 h after LPS injection (20 mg/kg) [53]. Taillé et al. observed a similar effect after one day from LPS administration [54]. These differences in results may stem from differences in tissue type and location of its collection. Nevertheless, this is the first study to present enhanced HO-1 levels following BQ123 administration in endotoxemic rats.

The second major enzyme defending mammalian cells against free radicals is SOD, which is known to occur in three isoforms: cytoplasmic SOD-1 (Cu/ZnSOD), mitochondrial SOD-2 (MnSOD), and extracellular EcSOD. The SOD family catalyzes the dismutation of potentially toxic superoxide anion to hydrogen peroxide (H_2_O_2_) and oxygen (O_2_) [55]. Recent studies indicate that SOD-1 expression is upregulated by Nrf2 pathway in a similar way to HO-1 [56, 57].

In the present study using rat femoral muscle, while injection of LPS or BQ123 alone had no effect on SOD-1 concentration, concomitant LPS and BQ123 administration resulted in significantly increased SOD-1 level. Such enhanced level of SOD-1 may increase the capacity of myocytes to diminish the raised superoxide anion level generated after LPS administration. Likewise, other authors also report unaltered SOD-1 levels after LPS treatment. Visner et al. have shown such effect in rat pulmonary artery and microvascular endothelial cells [58]. Liu et al. also report unchanged SOD-1 values 24 hours after treating gingival fibroblasts with 5 to 50 mg/mL of LPS [59]. In other studies, LPS had no influence on SOD-1 mRNA level in rat kidney [60], astrocytes [61], and human epithelial alveolar and airway cells [62, 63]. On the other hand, some authors report a reduction [57, 64] or increase [65, 66] of SOD-1 levels after LPS administration. Therefore, the SOD-1 results are ambiguous and require further study. So far, few authors demonstrated protective features of BQ123. Briyal et al. observed that BQ123 stimulated SOD production in the brain of amyloid-*β*-treated rat [25]. Moreover, BQ123 enhanced SOD activity, which was decreased after endothelin-1 (1-31) stimulation in rat cardiomyocytes [67]. Likewise, SOD activity was also increased after BQ123 treatment in myocardial ischemia-reperfusion injury [28]. However, Emre et al. indicate that BQ123 administration did not improve SOD activity in rat liver after renal ischemia-reperfusion injury [68].

Transcriptional factor Nrf2 is activated in response to inflammation and ROS. It plays a central role in the defense against them, since it controls the expression of detoxifying enzymes such as HO-1 and probably SOD-1. In the current study, LPS administration did not affect Nrf2 gene expression in rat skeletal muscle. Some authors have shown that LPS activates Nrf2 translocation to the nucleus in various tissues [52, 69], but few reports present Nrf2 gene expression after LPS stimulation. Hao et al. indicated unaltered level of Nrf2 mRNA in the murine heart after LPS challenge [70]. Yu et al. demonstrated an elevated level of Nrf2 mRNA in the lung of a rabbit model of endotoxemia [71], while Song et al. presented decreased Nrf2 expression in the diaphragm of preterm lambs after 72 h of LPS exposure* in utero *[72]. Similar research is needed on muscle tissue to clarify the occurring processes.

In this study, both pretreatment and treatment with BQ123 substantially raised Nrf2 mRNA level. What is more, elevated Nrf2 expression in the BQ123 + LPS group was associated with higher levels of HO-1 and SOD-1. Nrf2 is widely known to induce HO-1 production. Our data may indicate a signal path connecting Nrf2 and SOD-1. These data also suggest that BQ123 may be able to protect skeletal muscle cells from inflammation and oxidative stress through upregulation of Nrf2 expression, enhanced HO-1 and SOD-1 levels, and inhibition of RelA/p65 expression.
